# A three generation family with VACTERL association is found to have a rare form of diamond-blackfan anaemia

**DOI:** 10.1038/s41431-026-02076-z

**Published:** 2026-03-18

**Authors:** Iryna Leshchynska, Debjani Das, Victoria O’Reilly, Alena Sipka, Kavitha Iyer, Dimuthu Alankarage, Emma Rath, Akshita Kumar, Beth A. Kurt, Maria E. Voydanoff, The Congenital Heart Disease Synergy Study group, David Winlaw, Natasha Nassar, Edwin Kirk, Eleni Giannoulatou, Sally L. Dunwoodie, Roger E. Stevenson, David S. Winlaw, David B. Ascher, Eleni Giannoulatou, Paul R. Mark, Sally L. Dunwoodie, Gavin Chapman

**Affiliations:** 1 Victor Chang Cardiac Research Institute, Sydney, NSW Australia; 2 University of New South Wales, Sydney, NSW Australia; 3 School of Chemistry and Molecular Biosciences, University of Queensland, Brisbane, QLD Australia; 4 Corewell Health Helen DeVos Children’s Hospital, Pediatric Hematology Oncology, Grand Rapids, MI USA; 5 J. C. Self Research Institute of Human Genetics, Greenwood Genetic Center, Greenwood, SC USA; 6 Ann and Robert H. Lurie Children’s Hospital of Chicago, Chicago, IL USA; 7 Feinberg School of Medicine Northwestern University, Chicago, IL USA; 8 Computational Biology and Clinical Informatics, Baker Heart and Diabetes Institute, Melbourne, VIC Australia; 9 School of Clinical Medicine, Faculty of Medicine and Health, University of New South Wales, Sydney, NSW Australia; 10 Corewell Health, Helen DeVos Children’s Hospital, Medical Genetics and Genomics, Grand Rapids, MI USA; 11 Child Population and Translational Health Research, Children’s Hospital at Westmead Clinical School, Faculty of Medicine and Health, University of Sydney, Sydney, NSW Australia; 12 Centre for Clinical Genetics, Sydney Children’s Hospital, Randwick, NSW Australia; 13 Randwick Genomics Laboratory, NSW Health Pathology, Randwick, NSW Australia

**Keywords:** Disease genetics, Functional genomics

## Abstract

The spectrum of congenital malformations in VACTERL association varies among patients and can be differentially diagnosed with CHARGE syndrome, Fanconi anaemia, and others (reviewed in Solomon 2011). Despite overlapping clinical findings, the genetic causes of these diseases are distinct. In this context, unbiased whole genome sequencing can assist in differential diagnoses, as well as identify new gene-disease associations. In this report, we demonstrate that whole genome sequencing of a proband with suspected VACTERL association revealed two gene variants in the ribosomal genes *RPL18* (NM_000979.4 c.397 G > C p.(Gly133Arg)) and *RPS6* (NM_001010.3: c.370 C > G p.(Leu124Val)). Mutations in ribosomal genes are associated with Diamond-Blackfan anaemia, a condition that shares phenotypic similarities with Fanconi anaemia. Our modelling and functional assessment of the identified variants strongly indicate pathogenicity of the RPL18:p.(G133R) variant as it is novel, displays reduced expression and stability and abnormal intracellular distribution, and interferes with protein synthesis in cultured cells. The RPS6:p.(L124V) variant reduced protein expression and altered cytoplasmic distribution but does not interfere with protein synthesis in cultured cells. Overall, our study indicates the significant advantage of using unbiased whole genome sequencing for the examination of patients with complex congenital malformations.

## Introduction

VACTERL association describes the association of multiple congenital malformations: vertebral anomalies, anal atresia, cardiac defects, tracheo-eosophageal fistula, renal anomalies and limb abnormalities. VACTERL association is assigned when at least three of these malformations are present, and no clinical or laboratory-based evidence of an alternate diagnosis can be found. Differential diagnoses include but are not limited to CHARGE syndrome, Opitz G/BBB syndrome, Fanconi anaemia (FA) and others (reviewed in [1]) making their recognition based on clinical manifestation very challenging.

Recent advances in sequencing technology revealed different molecular mechanisms of these disease phenotypes. Currently, 19 genes are linked to VACTERL association phenotypes ([2], summarised on https://panelapp.genomicsengland.co.uk/panels/101/), 189 genes confidently linked to congenital heart disease (CHD) [3], 22 associated with FA [4] and 33 with Diamond-Blackfan anaemia (DBA) [5]. The necessity to analyse such a substantial number of genes makes it more reasonable not to restrict the genomic analysis to gene panels but rather take an unbiased approach and assess the patient’s entire genome. This approach permits evaluation of known disease-associated genes and enables the identification of new disease genes. Our recent work demonstrates the practicality and effectiveness of this approach by identifying in VACTERL association patients, mutations in novel disease genes *HAAO*, *KYNU* and *NADSYN1* as causes of Congenital NAD deficiency disorder (CNDD), and *WBP11* [6, 7].

In this report, we show that unbiased genetic testing of a proband with complex vertebral, heart, renal and endocrine phenotypes revealed variants in the ribosomal genes *RPL18*, previously linked to DBA [8], and *RPS6*. Functional assessments of identified variants indicate that both RPL18:p.(G133R) and RPS6:p.(L124V) proteins have reduced expression levels and abnormal intracellular localisation, but only RPL18:p.(G133R) exhibits reduced stability and interferes with protein synthesis in cultured cells. These data suggest that RPL18:p.(G133R) is functionally abnormal. Variants in ribosomal genes should be considered during the genetic diagnosis of patients with VACTERL association.

## Material and methods

### Clinical and genetic investigations

DNA was isolated from whole blood and sequenced using HiSeqX sequencing systems (KCCG Sequencing Laboratory, Sydney, Australia).

### Sequence alignment, variant calling, annotation of variant features and quality control

FASTQ sequencing data files were aligned to human reference genome GRCh38 without alternate contigs using bwa (version 0.7.17-r1188 [9]), followed by GATK best practices, including base quality score recalibration BQSR [10]. GATK (version 4.0.4.0 [10];) and Platypus [11] were used to joint-call SNPs, indels and multi-nucleotide variants (MNVs). Annotations of SNP/indel/MNV variants were carried out by ANNOVAR [12] and VEP (version 101[13]). Variant prioritisation was performed using VPOT [14]. Splicing variants, structural variants and copy number variants were also called and inspected.

### Variant curation

Variants with a minor allele frequency (MAF) less than 1% in the Genome Aggregation Database (v2 and v3 [15, 16]) were assessed. Variants were considered damaging if they—(a) altered the protein length, such as nonsense, frameshift, in-frame insertion/deletion and splice site variants, or (b) were missense variants with a REVEL [17] score ≥0.5 or a BayesDel [18] score ≥0.1 or a scaled CADD [19] score of ≥20, or (c) were structural variants in exonic regions. Predicted-damaging variants were verified by visual inspection of the variant locus using the Integrative Genomics Viewer (IGV [20]). Variants in highly polymorphic genes with notoriously high false-positive variant calls (e.g. mucins) were removed irrespective of read quality. An unbiased analysis was performed on IGV-verified coding gene variants for autosomal dominant (AD), autosomal recessive (AR), de novo and compound heterozygous inheritance models. MAF were set to <0.001 for AD inheritance models and <0.01 for AR models. Incomplete penetrance was considered for candidate variants shared by multiple individuals per family. Genes not previously associated with VACTERL association or CHD were assessed for their role in disease causality by manually curating the biomedical and life sciences literature using search engines including Mastermind (Genomenon, https://www.genomenon.com/mastermind/), PubMed (https://pubmed.ncbi.nlm.nih.gov) and disease-association databases including OMIM [21] and ClinVar [22].

The presence of *RPL18* and *RPS6* variants was verified by PCR and Sanger sequencing using forward/reversed primers CCAGTAGCCACACAGCTCAG/CCTCTCCACCAGGTATGTGC for *RPL18* and CTGGCGGACATCATCTTCTT/GCCAGTTGTCCAAATGCTTA for *RPS6*.

### Protein modelling

The structures of RPL18 (UniProt ID: Q07020) and RPS6 (UniProt ID: P62753) were extracted from the cryo-EM structure of the ribosome in complex with rRNA (PDB ID: 8GLP) [23], with a resolution of 1.67 Å. Chain AB and K correspond to RPL18 and RPS6, respectively, allowing us to isolate and analyse the specific interactions and conformational details for these proteins within the ribosomal complex. Arpeggio [24] was used to calculate atomic interactions in both the reference and variant structures, providing insight into changes in interaction patterns resulting from the variant. The impact of variants was evaluated using a suite of in silico structural stability and dynamic prediction tools. The effects of variants on protein stability were assessed using the state-of-the-art AI predictive tool DDMut [25], in addition to the statistical potential predictive tools INPS-3D [26] and MAESTROweb [27]. mmCSM-NA [28], a graph-based approach developed to predict the effects of variants on protein-nucleic acid binding affinity, was also used in order to evaluate the impact of variants on the interaction with rRNA. Additionally, the AlphaMissense [29] score and the Missense Tolerance ratio [30, 31] were used to provide further insight into each variant’s potential pathogenicity and purifying selective pressure, respectively.

### Cloning

The human *RPL18* (NM_000979.4) and *RPL18*:c.397 G > C gene fragments were synthesised (IDT, IA, USA). The cDNA encoding human *RPS6* (NM_001010.3) was amplified from a human cDNA library generated from iPSC cells with the SuperScript™ III First-Strand Synthesis System (ThermoFisher, MA, USA). Obtained DNA fragments and cDNAs were cloned into pEntr2B vector (ThermoFisher). The *RPS6*:c.370 G > C variant was introduced via site-directed mutagenesis. The gateway cloning system (ThermoFisher) was used to subclone reference and variant *RPL18* and *RPS6* into the pEF-EGFP-P2A-NLSRuby3-bGHpA vector for intracellular distribution and protein synthesis assays. For the stability assay, *RPS6*, *RPL18* and their variants were subcloned into pcDNA3-NFLAG, a gift from Susan Lindquist & Mikko Taipale (Addgene plasmid #87064) [32].

### Protein stability determination

HEK293T cells were maintained in DMEM (ThermoFisher) supplemented with 10% foetal calf serum (Cytiva, MA, USA) in 10% CO_2_ at +37 °C. Cells were transfected with cDNA encoding the FLAG-tagged variants together with GFP using Lipofectamine LTX (ThermoFisher) according to the manufacturer’s instructions. After treatment with either DMSO (control) or cycloheximide (Sigma Aldrich, MO, USA) for 6-8 h, cells were placed on ice, washed with cold PBS and lysed for 30 min with cold HENG buffer (20 mM Hepes-KOH pH 7.9, 150 mM NaCl, 2 mM EDTA pH 8.0, 20 mM sodium molybdate, 0.5% Triton X-100, 5% glycerol, protease inhibitors cocktail cOmplete (Roche, Switzerland) and 0.2 mM PMSF). Cell lysates were transferred into FLAG M2-antibody (Sigma-Aldrich) coated 96-well plates and incubated at +4 °C for 3 h. After washing with cold HENG buffer and PBS, plates were incubated with FLAG M2-antibody-HRP (Sigma-Aldrich, MO, USA) conjugates in PBS supplemented with 0.05% Tween20 for 1.5 h at room temperature. The activity of Horseradish Peroxidase was analysed using SuperSignal ELISA Pico Chemiluminescent Substrate (ThermoFisher). GFP fluorescence and chemiluminescence were detected using PHERAstarFS (BMGLabtech, Germany). All obtained chemiluminescent values were normalised to GFP fluorescence. Stability was calculated as ratio of cycloheximide treated cell lysate values to values obtained from control cell lysate. Unpaired t test was performed using Prism9 (GraphPad, CA, USA).

### Evaluation of protein expression and intracellular distribution

HeLa cells were maintained in DMEM (ThermoFisher) supplemented with 10% foetal calf serum (Cytiva, MA, USA) in 10% CO_2_ + 37 °C. Cells were transfected with Lipofectamine LTX (ThermoFisher) using the reverse transfection method according to the manufacturer’s instructions and plated in 96-well CellCarrier Plates (PerkinElmer, MA, USA) at lower density. After 24 h, cells transfected with RPL18 and RPS6 constructs were fixed with 4% PFA, washed with PBS, labelled with CF350 WGA (Biotum, CA, USA). Cells were washed twice with PBS and imaged with Opera Phenix (PerkinElmer, MA, USA), first at 10× to identify the transfected cells, and then at ×63 magnification in confocal mode to acquire images for analysis. Images of transfected cells were analysed using the Harmony® high-content analysis software v3.5 (PerkinElmer, MA, USA). NLS-Ruby positive HeLa cells were identified, imaged and sorted according to their NLS-Ruby fluorescence intensities. To control for variation in plasmid DNA transfection, GFP fluorescence intensity was analysed in cells with NLS-Ruby intensities below the sum of its median and mean absolute deviation. Level of expression, morphological and textural parameters were quantified. Statistical analysis was performed using in-house software. The normalised differences between variant and reference proteins were compared using one-sample t-test in Prism 9 (GraphPad, CA, USA).

### Analysis of protein synthesis

The efficiency of protein synthesis was analysed using SUnSET assay as described before [33]. Briefly, low density HeLa cell cultures were transfected with GFP-variant fusions and treated with 1 μM of puromycin (Sigma Aldrich, MO, USA) for 10 min in a CO_2_ incubator, washed with PBS and fixed with 4% of PFA. Puromycin incorporated into protein was detected with anti-puromycin antibody (Developmental Studies Hybridoma Bank, IA, USA) and visualised with Dylight647 secondary antibody (Jackson ImmunoResearch, PA, USA). Images of transfected cells were acquired and analysed using the Harmony® high content v3.5 (PerkinElmer, MA, USA) and Fiji analysis software. Protein synthesis was analysed in cells with NLS-Ruby intensities below the sum of its median and mean absolute deviation.

## Results

### Summary of relevant family history

The proband was born with vertebral and rib anomalies, atrial septal defect, coarctation of the aorta, absent right kidney, absent right thumb, duplicated left thumb, congenital hypothyroidism and foetal growth restriction with subsequent short stature.

The proband’s mother was born with a bifid thumb, extra vertebrae and fused coccyx. Maternal adult height of 150 cm (4’11”) is more than one standard deviation below her mid-parental predicted height. The proband’s maternal grandmother had Klippel-Feil syndrome with cervical fusion anomalies and Sprengel deformity. One living maternal aunt also has an incidental finding of an extra cervical vertebrae. Three affected family members have a diagnosis of Hashimoto’s thyroiditis without known congenital hypothyroidism as seen in the proband. A maternal uncle was born full-term with bifid thumb and reported congenital cardiac disease with suspected pulmonary haemorrhage resulting in death shortly after birth.

From a haematologic perspective, the proband had normocytic anaemia repeatedly identified between the ages of 30 months and 6 years with two episodes requiring transfusion for haemoglobin less than 70 g/L; after age 6 years, anaemia spontaneously resolved. He developed intermittent leukopenia and moderate to severe neutropenia starting at 12 months of age, but this was attributed to recurrent viral infections, and he was not referred to haematology clinic until age 30 months. He was treated initially with growth-colony stimulating factor (G-CSF) 1–2 times weekly from ages 3–6 years, then only intermittently in the setting of acute febrile neutropenia. At the age of 15 years, in addition to neutropenia and leukopenia, bone marrow analysis revealed low cellularity for age, a predominance of erythroblasts, and low myeloid-to-erythroid (M/E) ratio, suggesting ineffective erythropoiesis (Table 1) [34].

**Table 1:** Hematopoietic parameters observed in the proband at 15 years of age.

Lab tests	Proband’s values	Normal range
Bone marrow		
Cellularity	30 - 40% cellularity with erythroid predominance maturing trilineage haematopoiesis, no dysplasia	~85% cellular
Karyotype	46, XY	
500-cell manual differential count on the aspirate smear (500 cells)	24% myeloid cells, 56% erythroid cells, 13% lymphocytes, 4% plasma cells, 3% blasts.	
Myeloid/erythroid (M/E) ratio	0.4:1	2:1 to 4:1
Peripheral blood		
White blood cell count	1.7 × 10^9^/L	4–10.8 × 10^9^/L
Absolute neutrophil count	0.41 × 10^9^/L	1.800–7.800 × 10^9^/L
Haemoglobin	152 g/L	140–180 g/L
Haematocrit	41.2%	42.0–52.0%
Mean Cell Volume	83.6 fL	80.0–100.0 fL
Platelet	181 × 10^9^/L	140–400 × 10^9^/L
Reticulocyte %	1.44%	0.48–2.22%
Reticulocyte Absolute	71 × 10^9^/L	40–100 × 10^9^/L
Erythropoietin (EPO) level	17.0 mU/mL	4.5–29 mU/mL
Erythrocyte adenosine deaminase	567 mU/g Hb	400–900 mU/g Hb
Haemoglobin A	96.7%	95.0–98.0%
Haemoglobin F	<1.0%	<1.0%
Haemoglobin A2	3.0%	1.5–3.5%
Variant haemoglobin	0.0%	0.0%

Multiple family members demonstrate similar hematologic anomalies. Specifically, the maternal grandmother, mother, maternal aunt (II.4) and a first cousin have a history of leukopenia and mild to moderate neutropenia in the absence of acute illness. Family members with neutropenia have not undergone bone marrow aspiration nor have they required G-CSF, as was indicated for the proband.

### *RPL18* and *RPS6* variants identified by whole genome sequencing

**Fig. 1:**
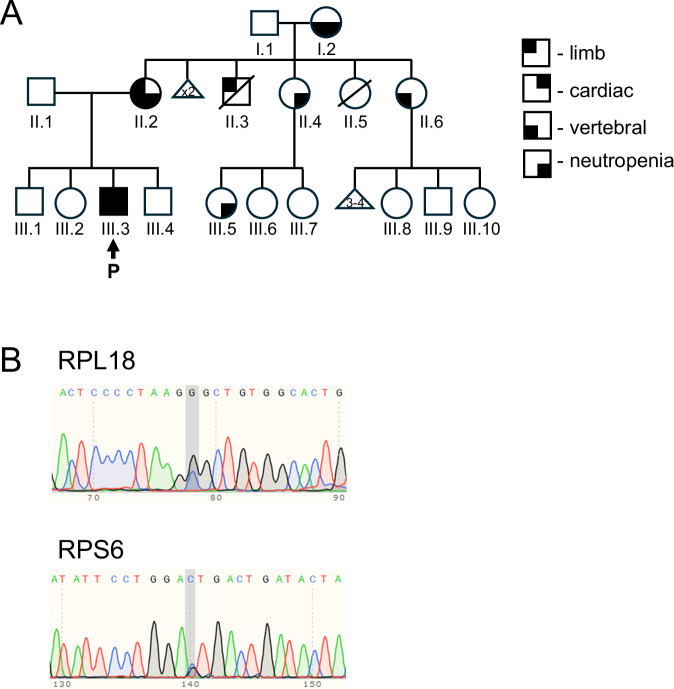
RPL18 and RPS6 variants identified in the proband with VACTERL association. **A** Pedigree of the family. P indicates proband (III.3). Black indicates some phenotypic features. Whole genome sequencing was performed on whole blood DNA from the proband (III.3) and III.4, II.1, II.2 and I.2. **B** Sanger sequencing electropherograms show *RPL18*:c.397 G > C and *RPS6*:c.370 C > G variants in proband genome sequence. The variant sites are highlighted in grey.

Whole genome sequencing (WGS) was completed for five family members: proband (III.3), unaffected father and brother (II.1 and III.4), and affected mother and maternal grandmother (II.2, I.2) (Fig. 1A). An unbiased comprehensive analysis of rare and predicted-damaging variants identified through WGS revealed missense variants in two ribosomal proteins encoding genes *RPL18*, *RPS6* and a glycosaminoglycan hydrolase encoding gene *IDUA* (Table 2). The proband was heterozygous for all three variants. No predicted damaging variants were identified in known CHD genes nor those that cause VACTERL phenotypes. The paternally inherited missense *IDUA* variant NM_000203.5 c.653 T > C p.(Leu218Pro) was classified as Pathogenic in ClinVar for recessive mucopolysaccharide conditions, including Hurler syndrome [35] (Table 2). The IDUA:p.(L218P) did not co-segregate with the disease phenotype and was not considered a likely casual variant for the proband (Fig. 1A and Table 3). The *RPL18* NM_000979.4 c.397 G > C p.(Gly133Arg) and *RPS6* NM_001010.3: c.370 C > G p.(Leu124Val) variants were maternally inherited, absent in the unaffected siblings (Table 3) and selected for further analysis. WGS findings were validated by Sanger sequencing (Fig. 1B) of the genome sequenced individuals and additional family members: proband’s sister (III.2), brother (III.1), maternal aunts (II.4 and II.6) and maternal grandfather (I.1).

**Table 2:** Variants identified in the proband by whole-genome sequencing.

Gene	Function	Genomic/Nucleotide/Protein variant	Inheritance	gnomAD	Pathogenicity prediction scores	Notes
*RPL18*^a^	ribosomal protein of the L18E family, component of the 60S subunit	NC_000019.10:g.48616103 C > G; NM_000979.4: c.397 G > C NP_000970.1:p.(Gly133Arg)	maternal, autosomal dominant	v2 = NA v3 = NA v4.1 = NA	CADD: 25.4 REVEL: 0.489 AlphaMissense: 0.9984	novel, predicted damaging variant in a haplo-insufficient gene, linked to Diamond-Blackfan anaemia
*RPS6*^a^	ribosomal protein of the S6E family, a component of the 40S subunit	NC_000009.12:g.19378494 G > C NM_001010.3: c.370 C > G NP_001001.2:p.(Leu124Val)	maternal, autosomal dominant	v2 = 5 Hets v3 = 4 Hets v4.1 = 33 Hets	CADD: 22.9 REVEL: 0.373 AlphaMissense: 0.4424	rare variant in a haploinsufficient gene
*IDUA*^a^	hydrolyses the terminal alpha-L-iduronic acid residues of the glycosamino-glycans, dermatan sulphate and heparan sulphate	NC_000004.12:g.1001742 T > C NM_000203.5: c.653 T > C NP_000194.2:p.(Leu218Pro)	paternal	v2 = 2 Hets v3 = 2 Hets v4.1 = 25 Hets	CADD: 26.2 REVEL: 0.918 AlphaMissense: 0.8387	rare and predicted damaging variant, classified as pathogenic in ClinVar, knockout mice have abnormal heart morphology [35]

^a^The proband is heterozygous for the variant.

**Table 3:** Association of *RPS6*/*RPL18* genotype with family members phenotype.

Individual	Relation to proband	Disease phenotype	RPS6:p.(L124V) genotype	RPL18:p.(G133R) genotype
I.1	Maternal grandfather	hypertension	heterozygous	homozygous reference
I.2	Maternal grandmother	Klippel Feil/Sprengel deformity medullary sponged kidney, Hashimoto’s thyroiditis, neutropenia	homozygous reference	heterozygous
II.1	Father	nil	homozygous reference	homozygous reference
II.2	Mother	extra vertebrae, bifid thumb, fused coccyx, cirrhosis, neutropenia	heterozygous	heterozygous
II.3	Maternal uncle	deceased, bifid thumb, pulmonary haemorrhage	na	na
II.4	Maternal aunt	neutropenia	homozygous reference	heterozygous
II.5	Maternal aunt	deceased	na	na
II.6	Maternal aunt	extra vertebrae, Hashimoto’s thyroiditis	homozygous reference	heterozygous
III.1	Brother	nil	homozygous reference	homozygous reference
III.2	Sister	nil	homozygous reference	homozygous reference
III.3	Proband	Klippel Feil/Sprengel deformity, short stature, single kidney, absent right thumb, duplicated left thumb, previous coarctation of the aorta, atrial septal defect, hypothyroidism, neutropenia	heterozygous	heterozygous
III.4	Brother	nil	homozygous reference	homozygous reference
III.5	Maternal 1st cousin	neutropenia	na	na
III.6	Maternal 1st cousin	nil	na	na
III.7	Maternal 1st cousin	nil	na	na
III.8	Maternal 1st cousin	nil	na	na
III.9	Maternal 1st cousin	nil	na	na
III.10	Maternal 1st cousin	nil	na	na

*na* not applicable as DNA not sequenced.

The RPL18:p.(G133R) variant has not been reported previously and was present in all affected family members, who exhibit disease phenotypes ranging from a single congenital defect to multiple defects involving different organ systems (Table 3). The RPS6:p.(L124V) variant was present in two of five affected family members and the unaffected grandfather (Table 3). The RPS6:p.(L124V) variant has also been reported in 33 individuals without paediatric disorders in gnomADv4.1, suggesting that the L124V substitution is likely tolerated and unlikely to be the primary cause of complex congenital defects. Notably, both variants were present in only two individuals, the proband and the mother, with a more severe disease phenotype. Thus, segregation analysis suggests that the RPL18:p.(G133R) is the likely causal variant, and the RPS6:p.(L124V) variant could be acting as a modifier to enhance the damaging impact of the RPL18:p.(G133R) variant. Hence, both variants were assessed for their effect on protein function.

### Modelling RPL18 and RPS6 variants in silico

Human ribosomes consist of the small 40S and large 60S subunits. The 40S subunit, composed of the 18S rRNA and RPS proteins, is primarily responsible for initiation of protein synthesis and monitoring the complementarity of tRNA and mRNA in protein translation. The 60S subunit assembled of 28S, 5S, 5.8S rRNA and RPL proteins, catalyses peptide bond formation during protein synthesis.

**Fig. 2:**
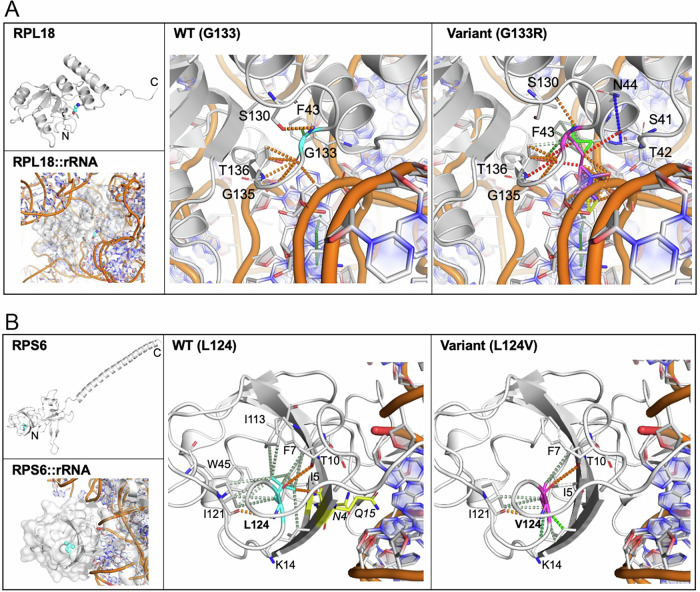
structure and molecular interactions in reference and variant RPL18 and RPS6. **A** RPL18 structure and molecular interactions in reference and G133R variant. (Top-Left) Cartoon representation of RPL18 with G133 residue highlighted in cyan sphere. The N- and C- termini are labelled for orientation. (Bottom-Left) Close-up view of the region of interest in RPL18, bound to rRNA (orange cartoon representation). The protein is shown in cartoon and transparent surface representations to emphasise spatial orientation; with G133 highlighted in cyan sphere. (Middle) Local interactions surrounding the reference G133 residue (cyan sticks), illustrating key contacts (residues highlighted in white sticks) that stabilise the position within the structure. (Right) Local interactions upon G133R (magenta sticks) mutation, highlighting intra- and inter-molecular interaction changes that could potentially impact protein stability and interaction with rRNA (orange cartoon representation). **B** RPS6 structure and molecular interactions. (Top-Left) Cartoon representation of RPS6 with the residue L124 highlighted in cyan sphere. The N- and C- termini are labelled for orientation. (Bottom-Left) A close-up view of the region of interest in RPS6, bound to rRNA (orange cartoon representation). The protein is shown in cartoon and transparent surface representations to emphasise spatial orientation; with L124 highlighted in cyan sphere. (Middle) Local interactions surrounding the reference L124 residue (cyan stick), illustrating key contacts (residues in white sticks) that stabilise the position within the structure. N4 and Q15 (adjoining I5 and K14) positioned at the rRNA-binding interface are highlighted in yellow sticks. (Right) Local interactions upon L124V (magenta stick) mutation, highlighting changes to intramolecular contacts that could potentially impact protein stability. The residue interaction patterns for reference and variant are illustrated as follows: hydrogen bonds are shown in red, polar interactions in orange, ionic interactions in yellow, amide-amide interactions in blue, carbonyl-van der Waals clashes in pink, aromatic interactions, including π-mediated interactions in dark green, and hydrophobic interactions in light green. The figures were prepared using Arpeggio [24] and PyMOL. A zoomed-in view of residues of interest is shown, with certain regions of protein and rRNA omitted for clarity, highlighting key interactions.

RPL18 is a component of the 60S subunit and the G133R variant is within the rRNA interaction domain (Fig. 2A). Qualitative analysis indicates that Glycine 133 is located in close proximity to the rRNA (Fig. 2A), and the change to arginine would result in the introduction of a much bulkier side chain, leading to local steric and electrostatic changes. Consistent with the disruption of intramolecular interactions, and broader steric changes, the G133R variant was consistently predicted to destabilise the RPL18 structure (Table 4). Interestingly, while the change to arginine would lead to steric clashes with the rRNA (Fig. 2A), it would also lead to a highly basic environment at the site, increasing rRNA-binding affinity (Table 4). AlphaMissense [29] assigned a high pathogenicity score of 0.99 and MTR3D [31] predicted this variant as intolerant (Table 4), supporting the likelihood of a damaging effect consistent with the predicted impact on protein stability and rRNA binding.

**Table 4:** Predicted structural and functional impact of RPL18:p.(G133R) and RPS6:p.(L124V).

Tools	RPL18:p.(G133R)	RPS6:p.(L124V)
Changes in variant protein stability		
*DDMut* [25]	−1.77 kcal/mol (Destabilising)	−0.73 kcal/mol (Destabilising)
*INPS-3D* [26]	−0.97 kcal/mol (Destabilising)	−1.61 kcal/mol (Destabilising)
*MAESTROweb* [27]	1.20 kcal/mol (Destabilising)	0.69 kcal/mol (Destabilising)
Changes in binding affinity upon variation		
*mmCSM-NA* [28]	0.54 (Increased affinity)	−0.68 (Decreased affinity)
Pathogenicity prediction score and Purifying selective pressures		
*AlphaMissense* [29]	0.99 (Pathogenic)	0.54 (Ambiguous)
*MTR3D* [30, 31]	0.19 (Intolerant)	0.80 (Moderate tolerance)

RPS6 is a ribosomal 40S subunit component and L124 is within the C-terminal domain (Fig. 2B) that binds and stabilises rRNA within the ribosome. L124 primarily engages in hydrophobic interactions with the N-terminal region (Fig. 2B), contributing to the structural integrity of the 40S subunit. Valine’s shorter side chain in the L124V variant reduces hydrophobic interactions, compromising local stability (Fig. 2B). In line with this observation, the variant was consistently predicted to destabilise the RPS6 structure (Table 4). Notably, while L124V does not directly disrupt the RNA-binding interface, the side chain of L124 engages in hydrophobic interactions with the backbone atoms of I5 and K14. These residues are adjacent to N4 and Q15, which are at the nucleotide-binding interface (Table 4 and Fig. 2B). The proximity and network of interactions suggest that this variant could indirectly affect the interface by destabilising the local region, thereby decreasing rRNA binding affinity (Table 4). AlphaMissense [29] and MTR3D [31] also captured this potential impact with a score of 0.54 (ambiguous) and 0.8 (moderate tolerance), respectively (Table 4), reflecting the complex role of this variant in modulating protein function.

### Functional analysis of RPL18:p.(G133R) and RPS6:p.(L124V)

**Fig. 3:**
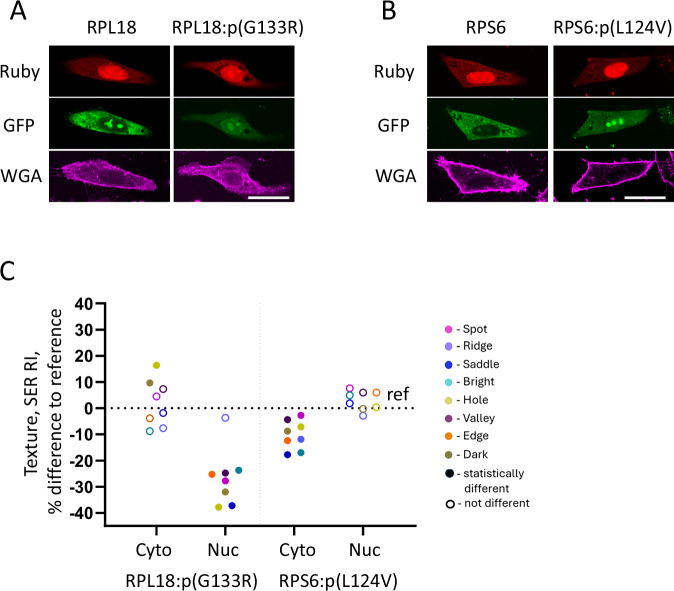
Intracellular distribution of RPL18 and RPS6 reference and patient variant proteins. RPL18 (**A, C**) and RPS6 (**B, C**) in cultured HeLa cells. **A**, **B** The proteins are expressed as GFP-fusions (shown in green), co-expressed nuclear localised ruby marks the transfected cells (shown in red), and the plasma membrane was visualised with wheat germ agglutinin (WGA) (shown in magenta). Scale bar 30 µm. **C** Texture analysis of reference and variant GFP-fusions expressed in cytoplasm (Cyto) and nuclei (Nuc) of cultured HeLa cells. The graph indicates the percentage difference in SER texture properties normalised to region intensity a 1 px scale (SER RI), and represented as the percentage difference observed in variant to reference proteins with reference set to 0. Circles highlighted with colours represent 8 calculated SER RI texture properties. The filled circles indicate statistically significant differences while hollow circles signify non-significant property differences. Individual data points represent the means of three independent experiments.

All GFP-tagged RPL18 and RPS6 reference and variant proteins expressed in HeLa cells, localised to the cytoplasm and nucleoli within the nucleus (Fig. 3A, B). For an in-depth assessment of the intracellular distribution of variant GFP-fusions, we analysed texture features using Spot, Edge, Ridge (SER) properties integrated in the Harmony software. Eight SER properties were calculated on GFP fluorescence intensity and normalised to the region’s average intensity to account for changes in texture due to possible differential expression of the variants.

In nuclei of RPL18:p.(G133R) expressing cells, seven SER texture properties were significantly reduced by more than 20% while RPS6:p.(L124V) did not alter textures in the nucleus compared to reference (Fig. 3C). RPS6:p.(L124V) exhibited a significant loss of all SER texture properties in the cytoplasm while cytoplasmic changes in RPL18:p.(G133R) texture were restricted to ‘Dark’ and ‘Hole’ properties, indicating the reduced patterning of RPL18:p.(G133R) in that region.

**Fig. 4:**
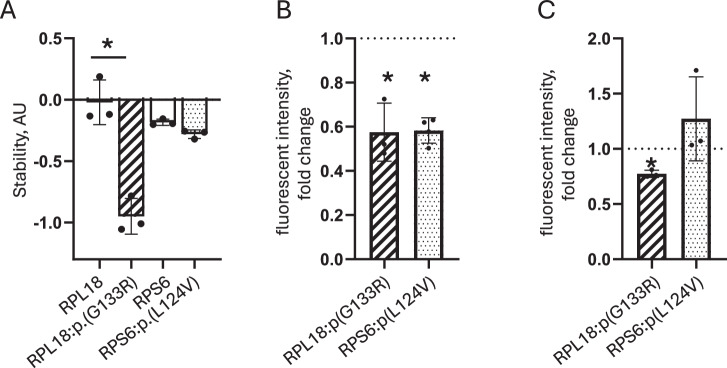
Functional analysis of variant proteins and RPL18:p(G133R) and RPS6:p.(L124V). **A** Stability of FLAG-tagged RPL18 and RPS6 variant proteins analysed by cycloheximide chase assay in the HEK cells. Graph shows means ± STD stability values relative to vehicle control. Bars represent the mean of three independent experiments. Individual data points are shown. *- statistically significant, *p* = 0.0023, unpaired t test. **B** Level of expression of reference and variant GFP-fusions in HeLa cells. The graph indicates the mean ± STD of fold differences in GFP normalised on co-expressed ruby fluorescence values. Individual data points indicating fold difference in three independent experiments are shown. The expression levels of reference proteins were set to 1 (dotted line). * - statistically different, *p* = 0.0259 (RPL18:p.(G133R)), *p* = 0.0111 (RPS6:p.(L124V)), unpaired t test. **C** Protein synthesis in HeLa cells expressing reference and variant GFP-fusions was analysed by SUnSET assay. The graph indicates the mean ± STD of fold differences in puromycin fluorescent intensity. The means of puromycin fluorescence intensities in cells expressing reference proteins were set to 1 (dotted line). Data points corresponding to three independent experiments are shown. * - statistically different, *p* = 0.0003 (RPL18:p.(G133R)), unpaired t test.

Changed intracellular distribution could be due to instability of proteins, which we analysed by cycloheximide-chase assay. On average 95% loss of RPL18:p.(G133R) versus 2% loss in reference RPL18 was observed after 6–8 h cycloheximide treatment, indicating significant instability of RPL18: p.(G133R). The stability of the RPS6:p.(L124V) protein was unchanged compared with RPS6 (Fig. 4A). Reduced stability can lead to insufficient protein levels. In HeLa cells, expression levels of RPL18:p.(G133R) and RPS6:p.(L124V) proteins were reduced by 42% and 34%, respectively (Fig. 4B).

The main function of ribosomes is protein synthesis. Reduced stability and misdistribution of ribosomal proteins can negatively impact translation and cause cell death [36]. We analysed translation efficiency in HeLa cells expressing the RPL18 or RPS6 GFP-fusion variant proteins using the SUnSET assay [33]. HeLa cells expressing variant GFP-fusions were treated with puromycin for 10 min, fixed and labelled with anti-puromycin antibody to assess the quantity of puromycin incorporated into the cellular proteins. Puromycin immunoreactivity in HeLa cells expressing the RPL18:p.(G133R) was reduced by 22% compared to reference RPL18 while it was unchanged between cells expressing RPS6:p.(L124V) and reference RPS6, indicating that the RPL18:p.(G133R) variant has a significant impact on translation (Fig. 4C).

## Discussion

In this study, we used whole genome sequencing to clarify the diagnosis of a proband with a complex congenital malformation phenotype and identified two maternal missense variants in genes encoding ribosomal proteins RPL18 and RPS6. Functional assessments and in silico modelling revealed that while both variants exhibit some differences to reference, only RPL18:p.(G133R) reduced the protein’s stability and had a detrimental effect on the efficiency of protein synthesis.

To our knowledge, no human disease associated with the *RPS6* gene has been reported. *RPL18* is associated with DBA18 (https://www.omim.org/entry/618310). The only other family described in the literature with an *RPL18* variant included a father with anaemia in infancy and persistent neutropenia, and a male child with anaemia and neutropenia. They were diagnosed with familial DBA, and neither had congenital anomalies [8].

The genotype-phenotype correlation is more complex in the family we report due to the presence of two variants in ribosomal protein genes. Four of the five RPL18:p.(G133R) carriers have neutropenia, indicating a diagnosis of DBA18. The maternal grandmother and maternal aunt II.4 carry only the RPL18:p.(G133R) variant. While maternal aunt II.4 has only neutropenia like the family reported in [8], the maternal grandmother has additional congenital anomalies suggesting variable expressivity of the RPL18:p.(G133R) variant. The proband and mother carry both *RPL18* and *RPS6* variants and have more complex malformations.

DBA is a ribosomopathy which is characterised by inherited bone marrow failure with subsequent cytopenias. The incidence of neutropenia in DBA is not widely reported. However, in one review that defined neutropenia as less than 1.5 × 10^9^/L, 14 of 35 (40%) of patients with genetically confirmed DBA had neutropenia. In addition, 17% of patients (6 of 35) had thrombocytopenia, suggesting that individuals with DBA may have more blood dyscrasias than anaemia. Although 98% of individuals with DBA present with anaemia in the first year of life [37], none of the members of the family presented with anaemia before age 30 months. Delayed presentation of anaemia has been reported in multiple patients with DBA [38], consistent with “non-classical” asymptomatic presentation of DBA [39].

Most variants in ribosomal genes associated with DBA with congenital malformations cause protein truncations resulting in the haploinsufficiency of ribosomal proteins [38, 40]. Haploinsufficiency is considered a major pathogenic mechanism of ribosomopathies [41]. Reduced cellular expression and significant instability of RPL18:p.(G133R) suggest a similar pathogenic mechanism. Deficiency in RPL18 was attributed to many negative effects like promotion of cellular senescence [42] or the appearance of pericardial oedema, decreased body length and subsequent death in zebrafish [43].

The molecular mechanisms of RPL18:p.(G133R) pathogenicity may be more complex than haploinsufficiency. The altered intracellular distribution of RPL18:p.(G133R) protein especially in the nucleus, where ribosomal subunits are assembled, supports this idea. Our multiple attempts to generate an RPL18:p.(G133R) mouse line failed, possibly due to embryonic lethality of potential founders.

Pathogenicity predictors and minor allele frequency suggest that the RPS6:p.(L124V) variant is not likely to be damaging. Despite changes to the cytoplasmic distribution and expression due to RPS6:p.(L124V), the variant protein did not interfere with protein synthesis in cultured HeLa cells. However, it remains possible that reduced expression of the RPS6:p.(L124V) compared to reference contributes to the patient phenotype. *Rps6* heterozygous null mouse embryos do not survive beyond embryonic day 5.5 due to cell cycle inhibition and induced apoptosis [44]. Depletion of *RPS6* in HeLa cells causes a significant decrease of mature 18S rRNAs, the component of the 40S ribosomal subunit, without affecting the maturation of 28S and 5.8S rRNA, the components of the 60S subunit responsible for the peptide bond formation [45]. Conditional depletion of *RPS6* in liver cells significantly reduces the ability of both immature and adult hepatocytes to survive and proliferate [46], raising the question of whether the cirrhosis observed in the mother might be due to a genetic predisposition. Likewise, conditional deletion of one *Rps6* allele in embryonic mouse limb bud mesenchyme results in underdevelopment or loss of bones of the limb and digits, with digits 1 and 5 most affected [47], a phenotype similar to absence of the right thumb in the proband.

In 2022, Luan et al. analysed gene expression and RNA translation after knockdown of every ribosomal subunit protein, including RPL18 and RPS6 in cultured A549 cells [42]. These data indicate that knock down of both *RPL18* and *RPS6* reduces translation of proteins involved in cell cycle inhibition and DNA damage response and increases translation of several proapoptotic genes. Depletion of RPS6 in cells also reduces the amount of other ribosomal proteins, including the constituents of the large 60S ribosomal subunit [45], which if it was to occur in the proband and mother could have exacerbated their phenotype. Further functional and expression studies of RPS6:p.(L124V) would be needed to establish any genetic modifier role the variant has in DBA.

Approximately half of patients with DBA have congenital anomalies including skeletal and thumb anomalies (18%), genitourinary malformations (11%) and CHD (12%) [48], while 75% of patients with Fanconi anaemia (FA) have physical features, with upper limb (35%), renal (20%) and congenital heart defects (6%) reported [49]. Additionally, up to 41% of patients with DBA [50] and 75% of patients with FA [50] have short stature, although questions remain about the impact of chronic steroid therapy and bone marrow transplantation on growth. Of interest, the two most common features prompting clinical geneticists to evaluate for FA are radial/limb anomalies and poor growth [51]. While chromosomal breakage studies were the traditional method to evaluate for possible FA, and more recently FANCD2 ubiquitination analysis [52], gene panels are now available for the diagnosis of both FA and VACTERL association. However, gene panels and gene studies for VACTERL association have not included DBA genes previously [53]. Although the likelihood of finding a genetic cause for VACTERL association is low, adding DBA genes to these panels and preferentially performing unbiased whole genome/exome sequencing should be strongly considered since identification of DBA early in childhood is critical for appropriate surveillance and treatment.

## Data Availability

The *RPL18* variant reported in this article is accessible in ClinVar with the submission number SUB15015230. The genomic datasets analysed during the current study are not publicly available due to ethical reasons. Raw experimental data can be made available upon request.
